# Efficacy of hc-tNGS for pathogen identification for pediatric cUTIs: a real-world observational study

**DOI:** 10.3389/fcimb.2026.1826277

**Published:** 2026-05-08

**Authors:** Lin Huang, Juanjuan Ding, Baolong Zhang, Jie Sun, Kai Chen, Na Li, Xiaoxia Peng, Huihui Yang, Xiaowen Wang

**Affiliations:** 1 Wuhan Children’s Hospital, Tongji Medical College, Huazhong University of Science & Technology, Wuhan, China; 2 Institute for Systems Biology, School of Life Sciences, Jianghan University, Wuhan, Hubei, China; 3 Beijing Children’s Hospital, Capital Medical University, Beijing, China

**Keywords:** hybrid capture-based targeted next-generation sequencing, pathogen identification, pediatric complicated urinary tract infection, pyelonephritis, vesicoureteral reflux

## Abstract

**Background:**

Complicated urinary tract infections (cUTIs) represent a critical subset of pediatric UTIs, characterized by diverse complex factors stemming from the patient, disease status, and pathogen-related attributes. Without timely and precise management, cUTIs may progress to invasive renal parenchymal infection (IRPI), posing long−term health risks. A central challenge in cUTI management lies in the limitations of urine culture, including prolonged turnaround time and suboptimal sensitivity, particularly post-antibiotic exposure. Hybrid Capture-Based targeted next-generation sequencing (hc-tNGS), a culture-independent emerging etiological assay, provides a potential solution to optimize clinical care. However, evidence regarding its utility in pediatric cUTI cohorts remains limited.

**Methods:**

This single−center, retrospective study enrolled 88 pediatric cUTI patients. All participants underwent sequential urine culture followed by hc−tNGS. The performance of the two methods in terms of pathogen spectrum, detection rate, and potential implications for antibiotic therapy optimization were compared.

**Results:**

Hc−tNGS demonstrated significantly higher pathogen detection sensitivity compared to conventional urine culture (92.5% vs. 33.0%, *p* < 0.001). Meanwhile, hc−tNGS uniquely identified polymicrobial bacterium-bacterium or bacterial-fungal infections in 58% cases, a profile missed by culture. Furthermore, *Klebsiella* species infection was identified as an independent risk factor for IRPI. Pathogen genetic profiling revealed that virulence and resistance genes were primarily clustered within *E. coli*-associated monomicrobial or polymicrobial pathogens, mainly including adhesion−related *ompA*, siderophore genes (*iucA/B/C*), and CTX−M resistance genotypes, respectively. In the vesicoureteral reflux (VUR) subgroup, this comprehensive profiling was associated with antibiotic adjustments in 71% cases.

**Conclusion:**

Sequential hc−tNGS provides a clinical diagnostic approach for cUTI as a complement to urine culture, especially after prior antibiotic use or suspected renal involvement. It enables accurate detection of polymicrobial and difficult−to−culture pathogens. These findings suggest that hc−tNGS may be integrated into routine diagnostic workflows to help guide targeted therapy and improve patient management in complex UTI settings.

## Introduction

Urinary tract infection (UTI) is one of the most common bacterial infections and a leading cause of fever during infancy. The annual incidence of UTIs has increased sharply, with affected individuals facing a substantial risk of progression to life-threatening renal abscess (Millner and Becknell, 2019; Simões et al., 2020; Kranz et al., 2024). Additionally, UTIs occurring in children aged <2 years serve as a sentinel event for underlying urologic developmental anomalies. Estimates of UTIs suggest that approximately 30% of children with congenital anomalies of the kidney and urinary tract (CAKUT), a condition affecting about 3–6 per 1000 live births, first present clinically with a UTI (Simões et al., 2020; Kolvenbach et al., 2023). Of these anomalies, VUR is clinically highly relevant to UTI risk, owing to its defining mechanism of retrograde urine flow from the bladder toward the kidney (Puri et al., 2024). In addition to patient-specific factors, indwelling urinary devices, immunosuppression, renal transplantation, and microbial attributes are also well-recognized risk factors for UTIs, thus constituting the cUTIs subgroup (Wagenlehner et al., 2020). Against this backdrop of complicating factors, cUTI is more challenging to manage and is often associated with poorer clinical outcomes compared with uncomplicated UTIs. Updated cUTI guidelines highlight that rapid, accurate pathogen identification during empirical antimicrobial therapy optimizes infection control and addresses the growing threat of antimicrobial resistance (Kranz et al., 2024). However, despite this clear clinical imperative, our understanding of the available methods to detect the spectrum of pathogens in pediatric cUTI and their diagnostic performance remains incomplete.

Conventional urine culture (UC) remains the gold-standard diagnostic. Its well-established reliability lies in characterizing isolates and performing subsequent antimicrobial susceptibility testing (AST). However, this method is inherently time-consuming, typically requiring 24–48 hours for microbial growth and an additional 24–36 hours to obtain susceptibility profiles (Chang et al., 2025). Moreover, urine culture exhibits low sensitivity to fastidious pathogens and often fails to accurately detect polymicrobial infections (Price et al., 2016; Nartey et al., 2024; Chang et al., 2025). This diagnostic limitation is particularly problematic in pediatric practice, as empirical antibiotic therapy is often initiated before successful urine sample collection, which significantly elevates the risk of false-negative results (Oreskovic and Sembrano, 2007). Such diagnostic constraints may consequently lead to misdiagnosis, inappropriate antimicrobial use, and poorer outcomes in pediatric cUTI management.

In this context, next-generation sequencing (NGS) has emerged as a promising diagnostic alternative, overcoming key limitations of culture-based methods by simultaneous high-throughput detection of a wide range of pathogens, antibiotic resistance genes, and virulence genes. Among its variants, metagenomic NGS has showed particular value for patients with culture-negative results (Jia et al., 2023; Benoit et al., 2024; He et al., 2024). Nonetheless, the cost associated with its unbiased sequencing of total nucleic acids and the required bioinformatic filtration of non-pathogenic background restrict its widespread use. In contrast, targeted NGS (tNGS), exemplified by hc-tNGS through probe-based enrichment, focuses sequencing on predefined pathogen panels, offering a more cost-effective and clinically feasible solution. Crucially, it sustains the practical utility of rapid pan-detection for a vast spectrum of known pathogens (Chen et al., 2024; Yin et al., 2024; Chang et al., 2025). Therefore, this technology is poised to become a vital complement to urine culture, necessitating assessment of its diagnostic accuracy, unique detection capabilities, and concordance with culture methods. To this end, this retrospective study collected clinical and microbiological data from cUTI cases with anatomical anomalies, poor response to empirical therapy, recurrent UTIs, or IRPI, all of whom had undergone sequential hc−tNGS testing after urine culture.

## Participants and methods

### Subjects

The pediatric patients with cUTIs who underwent sequential hc-tNGS after urine culture were recruited from Wuhan Children’s Hospital between August 1, 2024, and May 31, 2025. Since cUTIs often present as failure to respond to standard therapy, clinical classification systems exhibit slight variations in defining cUTI cases (Rubin et al., 1992; Hermanides et al., 2008; Flores-Mireles et al., 2015; Wagenlehner et al., 2020; McAteer et al., 2023). The present study primarily referenced the framework proposed by Wagenlehner et al.(Wagenlehner et al., 2020) for the enrollment of patients with cUTI. Specifically, pediatric patients were defined as having cUTI if they presented with at least one of the following complicating or risk factors: 1) rUTIs: defined as ≥ 3 episodes of UTI within one year or ≥ 2 episodes within the preceding 6 months (Kranz et al., 2024); 2) Breakthrough UTI (BT-UTI) in children with VUR but without meeting the diagnostic criteria for rUTI; 3) Poor response to empirical antimicrobial therapy (PREAT), with high risk of treatment failure: persistent recurrent fever despite 3-day empirical intravenous anti-infective treatment or 5-day sustained positive urine leukocytes; 4) IRPI confirmed by ultrasonography as acute focal bacterial nephritis (AFBN) or renal abscess. The exclusion criteria were: 1) parental refusal to perform hc-tNGS testing; 2) incomplete clinical data, specifically the absence of a valid urine culture result. The study has been approved by the Ethics Committee of Wuhan Children’s Hospital (Ethics Approval Number: 2024R141-E01). The research team did not interfere with any clinical diagnosis or treatment processes, and only retrospectively collected and analyzed anonymized clinical data from routine clinical care. Therefore, the requirement for informed consent for research participation was waived. Of note, hc−tNGS was performed as an external send−out test; written informed consent for the clinical test was obtained from legal guardians as required by hospital policy.

### Microbial culture and susceptibility testing

Clean-catch midstream urine samples were collected and processed according to the laboratory’s standard operating procedure, which is based on the WST489 standard for the diagnosis of urinary tract infections. After homogenization, 10 µL of urine was inoculated onto Columbia blood agar plates using the streak method and incubated at 37 °C with 5% CO_2_ for 24–48 hours. Interpretation of positive culture results was conducted in accordance with the Chinese Health Industry Standard “WST 489: Laboratory diagnosis of urinary tract infections”.

Traditionally, bacterial colony count ≥1×10^5^ CFU/mL has served as the classic threshold for UTI diagnosis. However, prior work has shown that in symptomatic patients, colony counts below this threshold can still signify clinically significant infections (Nelson et al., 2024). Thus, in this study, patients with colony counts between 1×10^4^ and 1×10^5^ CFU/mL for Gram-negative (G-) bacilli and between 1×10^3^ and 1×10^4^ CFU/mL for Gram-positive (G+) cocci were still classified as culture-positive, accounting for clinical symptoms and antibiotic use (lower counts likely due to antibiotic exposure). Isolation of the same microorganism in two consecutive samples with counts between 1×10³ and 1×10^4^ CFU/mL was also regarded as positive if contamination was ruled out.

AST was performed using the VITEK 2 Compact system (bioMérieux, France). Bacterial suspensions were adjusted to a 0.5 McFarland standard, loaded into AST cards, and incubated at 35 °C. Results (susceptible, intermediate, or resistant) were interpreted according to CLSI guidelines (M100-S35, 2025).

### hc-tNGS detection

**Figure 1:**
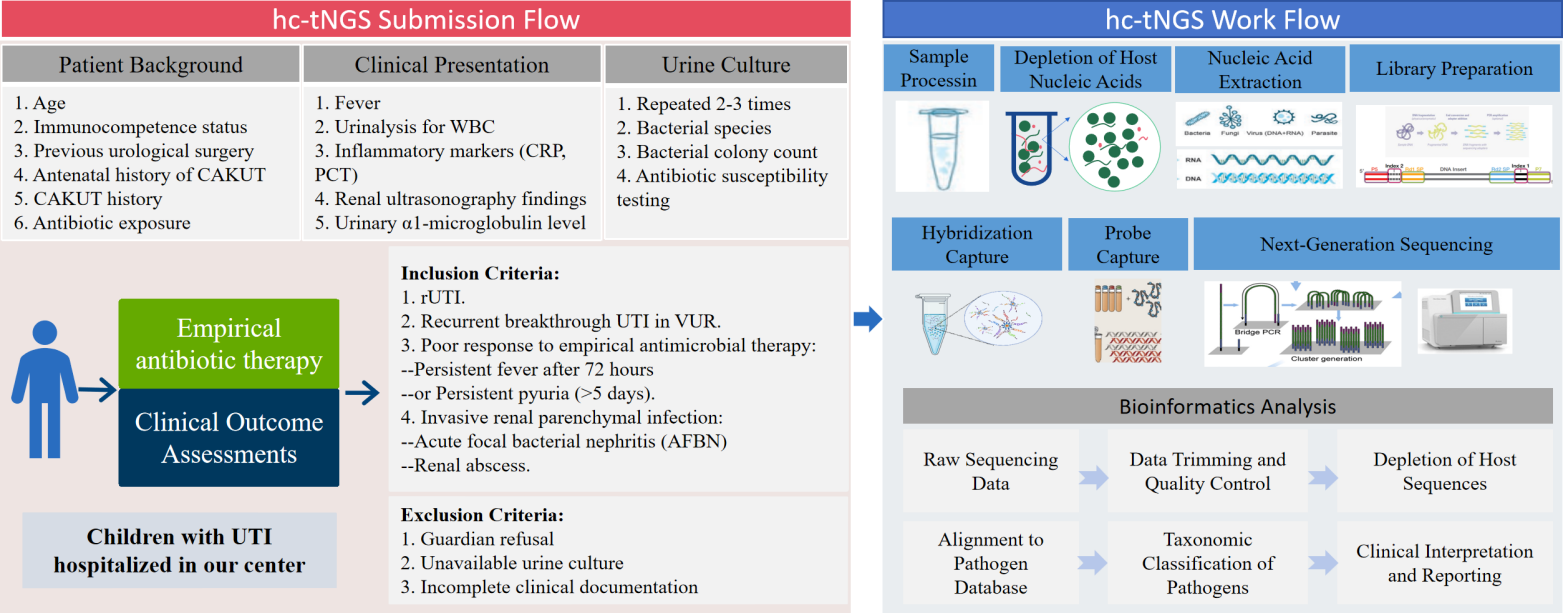
Flowchart of urine sequential hc-tNGS testing for cUTI in this study. The left panel outlines patient inclusion/exclusion criteria. The right panel shows the hc-tNGS laboratory process, from sample preparation to bioinformatic analysis. hc-tNGS, hybrid capture-based targeted next-generation sequencing; UTI, urinary tract infection; cUTI, complicated urinary tract infection; rUTI, recurrent urinary tract infection; CAKUT, congenital anomalies of the kidney and urinary tract; WBC, white blood cell; CRP, C-reactive protein; PCT, procalcitonin; VUR, vesicoureteral reflux; AFBN, acute focal bacterial nephritis.

The protocol for hc-tNGS of urine samples was primarily adapted from the method established by Yin et al (Yin et al., 2024). A 3-mL aliquot of each urine specimen was subjected to centrifugation for microbial enrichment, followed by the removal of host-derived cellular components. The processed pellets were then homogenized, and total nucleic acids (including DNA and RNA) of pathogenic microorganisms were extracted using the MataPure™ DNA&RNA Extraction Kit (KS121-WSWTO-48). Libraries were constructed through fragmentation, end-repair, adapter-ligation and PCR amplification. 8 uniquely barcoded libraries were pooled to hybridize and capture by specific biotinylated probes for 2h after library generation using the MetaBlood Pathogen Capture Metagenomic Assay Kit (KingCreate, China) and Qubit dsDNA HS Assay Kit (Thermo Fisher Scientific Inc.). Plasma-free nucleic acids and a fragmented human genomic DNA mixture were used as negative controls to detect contamination. A mixture containing inactivated bacteria (*Curtobacterium citreum*), fungi (*Schizosaccharomyces pombe*), and pseudovirus particles (bacteriophage MS2) was used as a positive control. Sequencing was performed on Illumina Miniseq platform setting to 100 bp single-end with average 1 million reads per sample. Sequence data was first processed by bcl2fastq software to demultiplex sequencing data and to convert base calling files into raw fastq-format files. Then Fastp was used to remove adapters, low quality sequences and duplicated sequences. The resulting data was aligned to human reference (hg38) using BWA and reads originated from human were filtered. After that, reads were compared to classification reference genomes database which contained 13,214 bacteria, 9,811 viruses, 3,180 fungi, and 405 parasites. Self-developed software was used to calculate the number of reads per million (RPM) for species or genus level and visualization of sequence alignment (**Figure 1**). The thresholds for bacteria, fungi, and viruses were 10, 4, and 6 RPM, respectively, established based on Yin et al (Yin et al., 2024), and applied to both polymicrobial and monomicrobial infections. Putative contaminant taxa (including common environmental or skin colonizers detected at low abundance without clinical correlation) were excluded from final pathogenic classification.

### Statistical analyses

Count data were expressed as frequency (percentage) [n (%)] and analyzed using the Pearson’s chi-square test or Fisher’s exact test (as appropriate). For multivariate binary logistic regression models employed to identify risk factors for invasive renal parenchymal infection (IRPI), candidate pathogens with a univariate *P* value < 0.05 were entered into the multivariate analysis. Odds ratios (ORs) and their corresponding 95% confidence intervals (CIs) were calculated. All statistical tests were two-tailed, and a significance level of α = 0.05 was set for all analyses. All analyses were conducted using SPSS 22.0 with a two-tailed *P*-value <0.05 considered statistically significant.

## Results

As a novel diagnostic technology, hc-tNGS was first implemented as a consecutive diagnosis following urine culture in our institution, due to concerns regarding medical costs and parental acceptability. The detailed submission flow and workflow of hc-NGS in this study are illustrated in **Figure 1**.

### Clinical characteristics

The cohort of cUTI in this study was predominantly composed of infants and toddler children. Of the 88 patients, 63 (71.6%) were aged ≤ 2 years, and 50 (56.8%) were males. Fever was observed as a clinical feature of UTI in 70 cases, accounting for 79.5%. From the perspective of infection frequency, 21 patients (23.9%) were diagnosed with rUTI and 56 patients (63.4%) had their first UTI episode. The remaining 11 patients had BT-UTIs, all receiving prophylactic antibiotics but failing to satisfy the diagnostic criteria for rUTI. When stratified by infection severity, 24 patients classified into the IRPI (14 cases of acute focal bacterial nephritis (AFBN) and 10 of renal abscess) uniformly presented with recurrent fever as their primary complaint. The other 64 patients, diagnosed with acute pyelonephritis (APN) or lower UTI, had 71.8% presenting with fever as the main complaint.

When assessing renal and urinary tract structural abnormalities, CAKUT were the predominant diagnosis (43/88, 48.9%). Among these, four patients had two distinct CAKUT phenotypes complicated with VUR, the primary CAKUT subtype of this cUTI cohort (31/88, 35.2%). These 31 VUR patients included 15 with prior VUR and 16 newly diagnosed cases. Among the non-VUR cases, 23 were confirmed by voiding cystourethrogram. The remaining 34 did not undergo VCUG, either due to parental refusal or a clinical assessment of negligible VUR risk (**Table 1**). Other documented structural anomalies included duplex kidney (7/88, with two featuring VUR), neurogenic bladder (4/88), terminal ureterocele (2/88), anterior urethral valve (status post-surgical repair, 2 cases with one featuring VUR), posterior urethral valves (PUV, 1 case with featuring VUR) and unilateral multicystic dysplastic kidney (MCDK, 1/88). Additionally, urinary tract stones (4/88) and autosomal dominant polycystic kidney disease (ADPKD; 2/88) were also identified.

**Table 1:** Comparison of pathogen positivity rates between urine culture and hc-tNGS in 88 pediatric patients with cUTI.

Clinical characteristics^a^	UC (n=29)^b^	tNGS (n=85)^b^
age ≤ 2yr (n=63)	26 (41.2%)	62 (98.4%)
age>2yr (n=25)	3 (12.0%)	23 (92.0%)
male (n=50)	13 (26.0%)	49 (98.0%)
female (n=38)	16 (42.1%)	36 (94.7%)
rUTI (n=21)	8 (38.0%)	21 (100%)
BT-UTI (n=11)	6 (54.5%)	10(90.9%)
initial UTI (n=56)	15(26.7%)	54 (96.4%)
APN and lower UTI (n=64)	24 (37.5%)	61 (95.3%)
AFBN and renal abscess (n=24)	5 (20.8%)	24 (100%)
VUR (n=31)	14 (45.1%)	29 (93.5%)
no VUR detected (n=23)	6 (26.0%)	22 (95.6%)
refused or incomplete VUR (n=34)	9 (26.4%)	34 (100%)
full-dose antibiotic (n=77)	23 (29.8%)	74 (96.1%)
prophylactic antibiotic (n=7)	4 (57.1%)	7 (100%)
no antibiotic therapy (n=4)	2 (50.0%)	4 (100%)
Number of detected pathogens		
bacteria	27 (30.7%)	85 (96.6%)
fungi	2 (2.3%)	9 (10.2%)
virus	0 (0%)	24 (27.3%)
mycoplasma and protozoa	0 (0%)	6 (6.81%)

a In this column, the n value in parentheses for each characteristic denotes the number of cases corresponding to that subgroup within the entire study cohort.

^b^The n value represents the number of cases with pathogen positivity identified by the respective method.

### Urine culture testing

**Figure 2:**
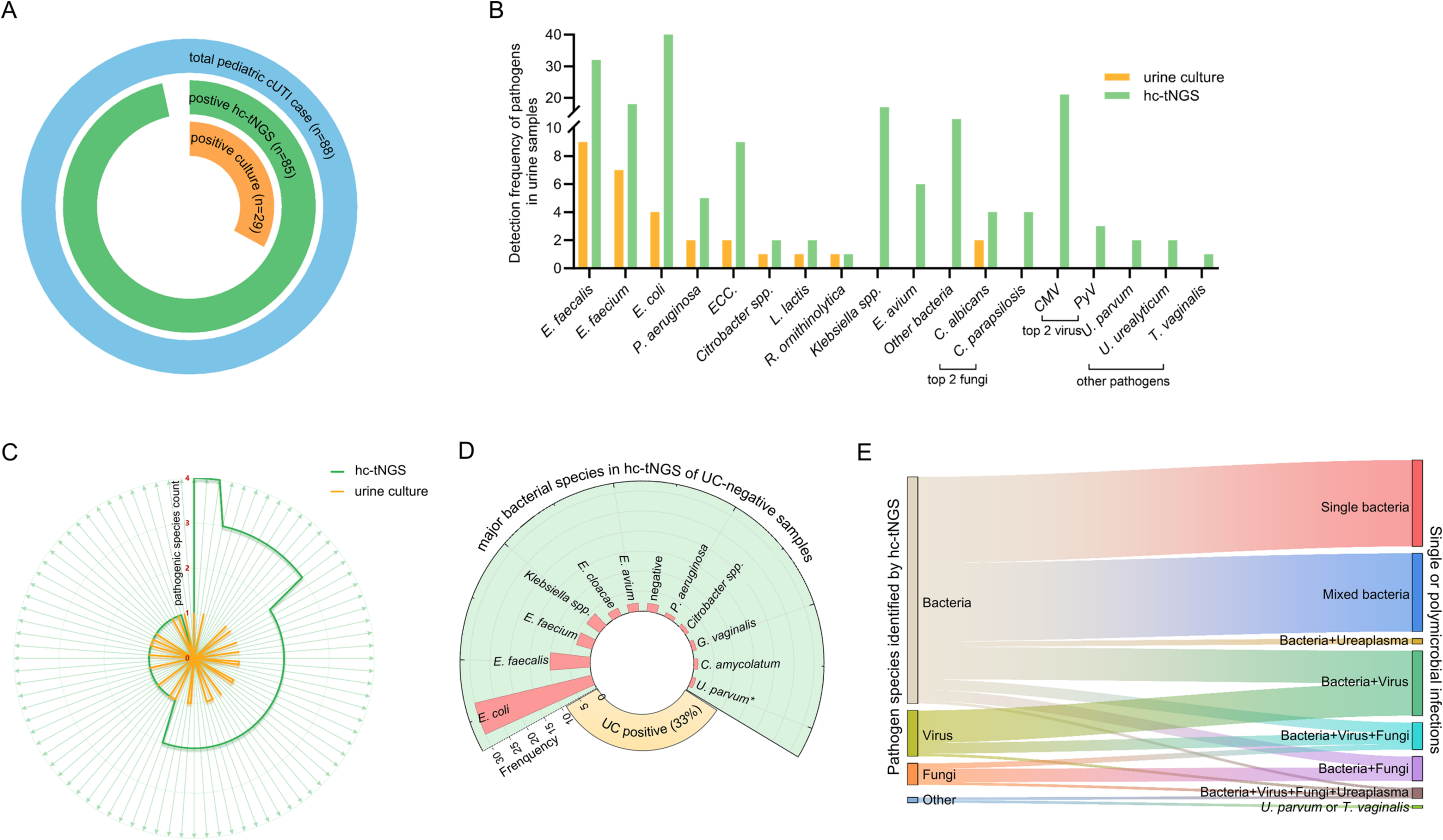
Pathogen detection performance of urine culture and hc-tNGS in pediatric cUTI. **(A)** Overview of positive results from urine culture and hc-tNGS across all 88 cUTI cases, illustrating the proportion of positive results. **(B)** Bar chart comparing the detection frequency of pathogens between urine culture (orange) and hc-tNGS (green), demonstrating that hc-tNGS exhibits higher sensitivity and a broader detection range. **(C)** Radial plot displaying the pathogen spectrum, with orange representing single pathogens detected by urine culture (29/88 samples) and green representing the broader detection range of hc-tNGS, where 36, 35, 11, and 3 samples were found to contain 1, 2, 3, and 4 known pathogen bacterium-bacterium or bacterial-fungal species, respectively. **(D)** Distribution of major bacterial species detected by hc-tNGS in samples with UC-negative cases (67%), showing the relative frequency of each pathogen. *Pathogens marked with an asterisk are non-bacterial. **(E)** Sankey diagram depicting the diversity of microorganism profiles detected by hc-tNGS, including single bacteria, mixed bacteria, and bacteria co-occurring with viruses, fungi, or other pathogens.

The urine culture positivity rate was relatively low at 33% (29/88) in this study (**Figure 2A**), which may be attributed to the prevalent use of antibiotics prior to sample collection (84/88; 95.5%) (**Table 1**). The main isolates were *Enterococcus faecalis* (*E. faecalis*; 9 cases), *Enterococcus faecium* (*E. faecium*; 7 cases), and *Escherichia coli* (*E. coli*; 4 cases); followed by *Pseudomonas aeruginosa* (*P. aeruginosa*) and *Enterobacter cloacae complex* (*ECC.*) (2 cases each). Rare pathogens included *Citrobacter species*, *Leuconostoc lactis*, and *Raoultella ornithinolytica* (1 case each) (**Figure 2B**). In addition to bacterial pathogens, urine culture from 2 patients yielded only the fungal pathogen *Candida albicans* (*C. albicans*), with no bacterial pathogens identified, including one postoperative case (anterior urethral valve repair) and one case of AFBN. Meanwhile, no polymicrobial bacterium-bacterium or bacterial-fungal infections were identified by urine culture (**Figure 2C**).

A subgroup analysis demonstrated that among patients with VUR, the urine culture positivity rate was 45.2% (14/31), higher than that in non-VUR patients (26.3%, 15/57; *p* = 0.072). Additionally, the positivity rate was 37.5% (24/64) in the group with APN or lower UTI, which was higher than that in the IRPI group (20.8%, 5/24; *p* = 0.138). In addition, the rate in patients with rUTI was slightly higher at 38.1% (8/21) compared with non-rUTI patients (26.8%, 15/56; *p* = 0.278). Notably, comparisons among the three subgroups only revealed a trend toward differences without reaching statistical significance, likely attributed to the relatively small sample size.

### Hc-tNGS testing

Hc-tNGS detected pathogenic bacteria in 85 patients (96.6%), covering 96.5% (28/29) of urine culture-positive cases and 96.6% (57/59) of urine culture-negative cases (**Figure 2A**). For the former, the pathogen detection concordance rate (consistent or inclusive) between the two methods was 96.6% (28/29). The only discordant case had a negative hc-tNGS result. In culture-negative specimens, the predominant bacterial strains in hc-tNGS reports were *E. coli*, *E. faecalis*, *E. faecium*, and *Klebsiella species* (*Klebsiella* spp.) not isolated by our urine culture (**Figure 2D**). Importantly, unlike urine culture, which predominantly indicated single pathogens, hc-tNGS exhibited remarkable capability in identifying polymicrobial bacterium-bacterium or bacterial-fungal infections (58%, **Figure 2C**, **Supplementary Figure 1**). More broadly, regardless of the pathogenic potential of individual taxa, mixed infection patterns in this study also included bacteria combined with other microorganisms, such as virus, fungi, *Ureaplasma* and protozoa (**Figure 2E**). Among these, cytomegalovirus (CMV) and polyomavirus (PyV) were the major viral pathogens, while *C. albicans* and *Candida parapsilosis* (*C. parapsilosis*) were the predominant fungal species detected in concurrent infections (**Figure 2B**). However, detection of these microorganisms, particularly viruses, does not necessarily imply clinical causation, especially in the immunocompetent hosts within our study. Notably, all 9 patients with bacteria-predominant polymicrobial infections involving fungi were diagnosed with rUTI. The implicated fungi included *C. albicans*, *C. parapsilosis*, and *Candida glabrata* (*C. glabrata*), while the major pathogenic bacteria comprised *E. coli*, *E. faecium*, and *K. pneumonia*. Among them, 2 patients had *C. albicans* isolated by urine culture but were negative for bacterial growth. Both recovered after a 2−week course of oral fluconazole for antifungal combined with targeted antibacterial therapy. Another two children who tested positive for *C. albicans* in hc-tNGS did not receive antifungal treatment due to the absence of CAKUT. Their urinalysis could normalize with enhanced supportive care and antibacterial therapy alone. Additionally, four patients tested positive for *C. parapsilosis* by hc-tNGS, while their urine culture results were negative. Of these, two patients, one with renal abscess and one with multiple urinary tract calculi, showed persistently abnormal pyuria despite empirical antibacterial treatment before the hc−tNGS results were available. After adding a 2−week oral fluconazole, their urine leukocyte counts returned to normal. Similarly, the remaining *C. glabrata*-positive case with VUR presented with an identical clinical profile, with persistent pyuria. Fluconazole treatment subsequently yielded a favorable response.

A high viral load (sequence count ≥10³) was observed in 26 patients, including 23 with CMV and 3 with PyV. None of the patients in this study had a history of organ transplantation, immunosuppressant use, or immunodeficiency; therefore, no antiviral therapy was administered (Zhan et al., 2023). In addition to virus and fungi, six patients had other pathogens co-detected with pathogenic bacteria, primarily *Ureaplasma urealyticum* (*U. urealyticum*) and *Ureaplasma parvum* (*U. parvum*). Notably, low-sequence reads of *Trichomonas vaginalis* (*T. vaginalis*) were co-detected in one patient (**Figure 2B**). Given the absence of clinical evidence supporting active infection, these non-bacterial or non-fungal agents were regarded as colonizing or transient microorganisms rather than causative pathogens at the present study.

Among all 88 patients enrolled in this study, three had negative hc−tNGS results. Of these, two patients were also urine−culture negative: one had multiple urinary tract calculi, and the other had completed a 2−week course of antibiotic therapy at another hospital prior to sampling. The remaining child had negative hc-tNGS but positive UC for *E. faecium*. Since urine culture samples were collected 2 days before hc-tNGS, the negative hc-tNGS result was likely due to delayed sample collection after the infection had been controlled. In summary, compared with urine culture, hc-tNGS not only identified a more diverse spectrum of pathogenic microbes but also detected a broader range of bacterial species.

### Pathogenic spectrum and risk factors for IRPI

In this study, the IRPI group comprised 24 patients diagnosed with either AFBN or renal abscess, aged 1 month to 14 years and most with initial febrile UTIs (**Supplementary Figure 1**). The UC positive rate in this group was 20.8%, while the hc-tNGS reached 100%. Furthermore, hc-tNGS results indicated that 83.3% of patients had polymicrobial infections, predominantly involving Gram-negative bacteria. The pathogenic bacteria were ranked by detection frequency as follows: *E. coli* (n=10), *Klebsiella* sp. (n=9), *E. faecalis* (n=6), *E. faecium* (n=3), and *ECC.* (n=2). Less common pathogens, each identified in one case, were *P. aeruginosa*, *Proteus mirabilis*, *Corynebacterium afermentans*, and *Leuconostoc lactis*.

Clinically, IRPI is an incompletely addressed form of UTIs that may be underdiagnosed without adequate imaging studies. In our study, multivariate binary logistic regression was utilized to identify pathogen-associated risk factors for IRPI episodes. Among common pathogens, *Klebsiella* sp. infection emerged as an independent risk factor (OR = 6.43, 95% CI = 1.86-22.26, *p* = 0.004), despite being associated with a relatively wide confidence interval (**Table 2**).

**Table 2:** Risk factor analysis for invasive renal parenchymal infection by multivariate binary logistic regression.

Variable	OR	95% CI		*P* value
*Klebsiella* spp.	6.430	1.857	22.261	0.003
*E. faecalis*	0.338	0.103	1.105	0.073

### Virulence and resistance gene profiles in cUTI pathogens

**Figure 3:**
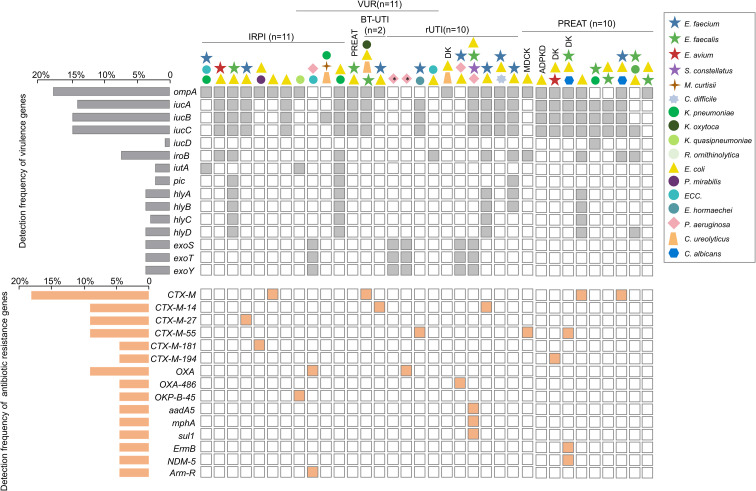
Virulence and antibiotic resistance gene patterns of pathogens identified by hc-tNGS. The horizontal gray bars on the upper left indicate the detection frequency of distinct virulence genes, while the horizontal orange bars on the lower left represent the detection frequency of various antibiotic resistance genes. Colored symbols at the top denote the pathogen combinations harboring the indicated virulence or resistance genes. Each symbol corresponds to a pathogen as defined in the right legend, with the vertical order of symbols indicating tNGS pathogen sequence abundance: the topmost symbol represents the dominant pathogen in each case. Clinical characteristics of cases with these pathogens and their associated virulence/resistance genes are described at the top. Symbols marked with an asterisk represent cases of surgical site infection-related healthcare-associated infections. One case (left) was associated with fenestration surgery for ureterocele complicated by hydronephrosis. The second case (right) was related to ureteral stent placement performed during surgical correction of VUR.

A total of 14 virulence genes were detected in 34 patients, predominantly including diverse chromosomally encoded virulence gene combinations from enterobacteriaceae infections. Among these, the adhesion/colonization-associated *ompA* and iron metabolism-related gene cluster (*iucA/B/C*) showed the highest detection frequencies and were frequently found to co-occur (**Figure 3**). Secretory hemolysin and exotoxin genes for potent cytotoxic effects, such as *hlyA/B/C/D* (*Enterobacter* spp.) and *exoS/T/Y* (*P. aeruginosa*), were detected at a relatively low frequency. Accordingly, these virulence genes were found primarily in infections (monomicrobial or polymicrobial) associated with *E. coli* (27/40 cases), *Klebsiella* spp. (7/16 cases), and *P. aeruginosa* (5/5 cases).

Resistance genes were identified in 17 patients and coexisted with the virulence gene *ompA*. CTX-M-type (cefotaxime-hydrolyzing enzymes, first isolated in Munich) were the most prevalent, mediating resistance to β-lactam antibiotics and predominantly associated with *E. coli*-associated infections (14/40 cases). OXA-type, another class of β-lactamases, were preferentially detected in *P. aeruginosa*-associated infections. Co-detection of multiple resistance genes in a single case was rare, and such cases were exclusively attributed to polymicrobial infections involving different bacterial genera. Notably, high-risk carbapenem resistance gene *NDM-5*was also identified in one case.

Focusing on 17 cases with detected resistance genes, only 5 cases had urine culture- positive results. In these cases, hc-tNGS demonstrated high concordance with culture for identifying causative pathogens and predicting resistance phenotypes. The genotypic identification of CTX-M-14, CTX-M-55, and OXA-486 was consistent with the phenotypic outcomes ofAST. For example, in case 2, tNGS detected CTX-M-14 in *E. coli*, which correlated with the AST result showing resistance to multiple cephalosporins (**Table 3**). In summary, enterobacteriaceae pathogens (e.g., *K. pneumoniae* and *E. coli*) in this study simultaneously harbored multiple virulence and resistance genes, probably serving as the core pathogens responsible for polymicrobial cUTI. In contrast, *Enterococcus* spp. pathogens (e.g., *E. faecium* and *E. faecalis*) were found to carry macrolide resistance genes (e.g., *ermB*) in only one case.

**Table 3:** Antimicrobial susceptibility of hc-tNGS-identified resistant pathogens in positive urine cultures.

Item Case	Case 1	Case 2	Case 3	Case 4	Case 5
Pathogen_tNGS_	*E. faecalis*, *P.aeruginosa*, *E. coli*	*E. faecalis*, *E. coli*	*E. coli*, *R.ornithinolytica*, *E. faecium*	*E.faecalis, E.hormaechei*	*P. saeruginosa*
ARG_tNGS_	*OXA-486*	*CTX-M-14*	*CTX-M*	*CTX-M-55*	*OXA*
Pathogen_UC_	*E. coli*	*E. coli*	*R. ornithinolytica*	*E.faecalis*,	*P. saeruginosa*
AST^a^	trimethoprim-sulfamethoxazole resistant	multiple cephalosporins^c^ and penicillins/β-lactamase inhibitors resistant	trimethoprim-sulfamethoxazole resistant	furadantin intermediate	meropenem intermediate; cefoperazone/sulbactam intermediate
Antibiotics^b^	ceftazidime; avibactam; fosfomycin	ceftazidime; teicoplanin	ceftaroline; amoxicillin-clavulanate	teicoplanin; piperacillin-tazobactam	piperacillin-tazobactam

UC, urine culture; AST, antimicrobial susceptibility testing;.

^a^Key resistant or intermediate results of AST;

^b^Final antimicrobial regimen based on tNGS and AST;

^c^For this case, the term “multiple cephalosporins” encompasses cefadroxil, cefalexin, cefradine, cefuroxime, cefaclor, cefamandole, cefoxitin, ceftriaxone, cefotaxime, cefdinir, cefoperazone and cefepime.

### Hc−tNGS’s impact on antibiotic adjustment in pediatric cUTI with VUR

The ultimate goal of pathogenic detection strategies, such as urine culture and hc-tNGS, is to inform optimal antibiotic therapy. However, therapeutic decisions for cUTIs are complex, relying on multiple clinical and microbiological indicators due to significant patient heterogeneity. Given this complexity, we focused the present analysis on a specific, high-risk population, patients with VUR, to evaluate the impact of hc-tNGS on antibiotic regimen adjustment.

In the high-risk VUR subgroup, hc-tNGS-derived microbiological profiles, when integrated with routine clinical evaluations, were associated with antimicrobial regimen adjustments in 71% of patients. These adjustments were mainly driven by findings such as culture-negative results, culture-uncovered drug-resistant bacteria, or the detection of previously culture-unidentified pathogens from a different bacterial family. Following hc-tNGS pathogen profiling, initial narrow-spectrum antibiotics were either escalated to broad-spectrum regimens, combined with more powerful anti-resistant agents, or supplemented with antibiotics targeting the newly identified bacterial family. Similarly, de−escalation from broad−spectrum antibiotics to narrow−spectrum agents, or discontinuation of one component from combination regimens, was also performed once hc−tNGS identified the clinically definitive causative pathogen. Notably, hc−tNGS results were never used in isolation; all adjustments were made at the treating clinician’s discretion, considering all available clinical and laboratory data. No antibiotic modification was implemented in the remaining 29% of cases, either due to concordance between UC and hc-tNGS results or as UC-identified pathogens represented the predominant pathogens in hc-tNGS-detected polymicrobial spectra, with the current antibiotic regimen exhibiting activity against the remaining pathogens. All patients achieved clinical resolution of infection after receiving standardized antibiotic courses following these adjustments. These observational findings suggest that hc−tNGS, when used alongside conventional diagnostics, may assist in targeted antibiotic adjustment for VUR patients via precise pathogen profiling.

## Discussion

cUTIs represent a prevalent clinical challenge in pediatrics, with recurrent episodes or progression to IRPI. Consequently, timely infection control and screening for underlying complicating factors form the cornerstone of cUTI management in children (Wagenlehner et al., 2020; Mattoo et al., 2021). Achievement of the former largely depends on the accurate identification of pathogenic microorganisms, which is challenging for conventional urine culture in cases of polymicrobial infection or when children have already received antibiotic therapy (Chang et al., 2025). In this retrospective study, we found hc-tNGS had a higher detection rate and marked advantages for identifying polymicrobial profiles, highlighting its potential to deepen our understanding of uropathogen ecology and advance precision clinical management.

Aligning with findings from other UTI populations, pathogenic *E. coli* remained the predominant causative agent in pediatric cUTI in our cohort based on hc-tNGS rather than urine culture (Millner and Becknell, 2019; Simões et al., 2020). This is likely owing to pre-collection antimicrobial exposure inducing *E. coli* to adopt a cell-wall-deficient (L-form) state, an alteration that impairs its growth on conventional media but potentially mediates immune evasion and bacterial persistence (Davison et al., 2020). The same observation was noted in *K. pneumoniae*, with urine culture repeatedly failing to identify it. Moreover, hc-tNGS results also indicated that mixed infections involving *E. coli* were significantly more common than monomicrobial ones. This complex pathogen profile likely reflected the intrinsic microbial complexity in cUTI patients. Mechanistically, certain polymicrobial infections may exert synergistic effects that exacerbate disease progression. For instance, *in vitro* studies have indicated that co-culture of *Enterococcus* and *Proteus mirabilis* can enhance biofilm biomass and mutually amplify antibiotic resistance (Timm et al., 2025). Additionally, mixed infection detections warrant cautious interpretation, as some positive microbial findings may represent colonization, transient shedding, or background signals rather than true causative pathogens. Accordingly, hc−tNGS results should be interpreted in conjunction with the clinical context rather than relying on sequence detection alone.

Both virulence and resistance genes were also frequently detected in infections involving *E. coli* (both monomicrobial and polymicrobial), underscoring a concerning trend of antimicrobial resistance development centered on this key pathogen. This trend has likely contributed to a distinct shift in the pathogen profile of IRPI. Whereas *P. aeruginosa* predominated a decade ago (Bitsori et al., 2015), our data now position *E. coli* and *Klebsiella* spp. as the two most prevalent pathogens. Meanwhile, *Klebsiella* spp. infection emerged as an independent risk factor for IRPI, a finding likely attributable to its virulence characteristics, such as pilus-mediated biofilm formation and high-level siderophore expression (Ambite et al., 2021). Notably, the limited sample size of IRPI cases in our cohort may have introduced bias and further validation in larger clinical cohorts is warranted.

VUR represents a major etiological factor for UTIs in the pediatric population (Chesney et al., 2008). To mitigate the risk of UTI recurrence in children with VUR, long-term prophylactic antibiotic therapy is frequently recommended. However, this strategy remains controversial, requiring careful balancing of therapeutic benefits against the risk of exacerbating antimicrobial resistance selection (Simões et al., 2020). One limitation of this study is that the small sample size and short observational period preclude a comparison of resistance gene profiles in VUR patients with versus without prophylactic antibiotic use. Expanding the cohort size, extending the study duration, and incorporating tNGS, could offer valuable insights into this question.

Furthermore, another limitations warrant acknowledgment. Firstly, constrained by new−technology validation and cost considerations, urine samples were submitted sequentially after culture, probably introducing a temporal discrepancy that may bias comparative analyses. To mitigate this, a standardized protocol for parallel submission will be implemented to enhance methodological rigor. Secondly, since the detection of microbial nucleic acids does not necessarily indicate definitive pathogenic activity, the clinical interpretation must take into account multiple factors, including the read count and the immune status of the host. Meanwhile, although pathogen identification remains central to stewardship, a comprehensive strategy for pediatric cUTI must also address appropriate antibiotic choice.

Collectively, our findings demonstrate that as a novel etiological tool, hc−tNGS aligns with the individualized and stratified management of cUTI, offering high sensitivity, broad pathogen coverage, and concurrent profiling of resistance and virulence markers. Computerized provider order entry (CPOE) systems are already employed to guide antibiotic selection based on patient and pathogen data (Gohil et al., 2024). While the present observational study cannot establish a definite causal relationship supporting the antimicrobial stewardship benefit of hc−tNGS, our findings support the feasibility of integrating molecular diagnostics into routine clinical decision−making. If prospectively validated in randomized controlled trials, tNGS results could be incorporated into CPOE systems alongside conventional clinical data to develop more precise decision−support models, thereby advancing targeted and precision antimicrobial stewardship.

## Data Availability

The original data presented in the study are included in the article/**Supplementary Material**. Further inquiries can be directed to the corresponding authors.

## Supplementary material

Supplementary Figure 1 Sequence counts (log_10_ scale) of positive bacteria and fungi in 85 hc-tNGS positive pediatric cUTI patients enrolled in this study, stratified according to the criteria for cUTI: breakthrough UTI (BT-UTI) **(A),** recurrent UTI (rUTI) **(B)**, poor response to empirical antimicrobial therapy (PREAT) **(C)**, and invasive renal parenchymal infection (IRPI) **(D)**. The x-axis (Case + number) indicates the patient identifier. Underlined case numbers (excluding the IRPI group) denote patients who also presented with IRPI. Different colored symbols represent distinct bacterial or fungal species. The black dot at the center of each symbol denotes the exact log_10_ value of the sequence count for the corresponding microorganism. *E. faecium, Enterococcus faecium; E. faecalis, Enterococcus faecalis; E. avium, Enterococcus avium; S. constellatus, Streptococcus constellatus; M. curtisii, Mobiluncus curtisii; C. difficile, Clostridioides difficile; K. pneumoniae, Klebsiella pneumoniae; K. oxytoca, Klebsiella oxytoca; K. quasipneumoniae, Klebsiella quasipneumoniae; K. variicola, Klebsiella variicola; R. ornithinolytica, Raoultella ornithinolytica; E. coli, Escherichia coli; P. mirabilis, Proteus mirabilis; ECC, Enterobacter cloacae complex; E. hormaechei, Enterobacter hormaechei; P. aeruginosa, Pseudomonas aeruginosa; C. ureolyticus, Campylobacter ureolyticus; C. albicans, Candida albicans; C. parapsilosis, Candida parapsilosis; C. glabrata, Candida glabrata; G. vaginalis, Gardnerella vaginalis; Citrobacter spp., Citrobacter species; S. aureus, Staphylococcus aureus; A. europaeus, Actinomyces europaeus; L. lactis, Lactococcus lactis; C. amycolatum, Corynebacterium amycolatum.*

## References

[B1] Ambite I. Butler D. Wan M. L. Y. Rosenblad T. Tran T. H. Chao S. M. . (2021). Molecular determinants of disease severity in urinary tract infection. Nat. Rev. Urol. 18, 468–486. doi: 10.1038/s41585-021-00477-x. PMID: 34131331 PMC8204302

[B2] Benoit P. Brazer N. de Lorenzi-Tognon M. Kelly E. Servellita V. Oseguera M. . (2024). Seven-year performance of a clinical metagenomic next-generation sequencing test for diagnosis of central nervous system infections. Nat. Med. 30, 3522–3533. doi: 10.1038/s41591-024-03275-1. PMID: 39533109 PMC11645279

[B3] Bitsori M. Raissaki M. Maraki S. Galanakis E. (2015). Acute focal bacterial nephritis, pyonephrosis and renal abscess in children. Pediatr. Nephrol. 30, 1987–1993. doi: 10.1007/s00467-015-3141-3. PMID: 26076753

[B4] Chang Z. Deng J. Zhang J. Wu H. Wu Y. Bin L. . (2025). Rapid and accurate diagnosis of urinary tract infections using targeted next-generation sequencing: a multicenter comparative study with metagenomic sequencing and traditional culture methods. J. Infect. 90, 106459. doi: 10.1016/j.jinf.2025.106459. PMID: 40058503

[B5] Chen Q. Yi J. Liu Y. Yang C. Sun Y. Du J. . (2024). Clinical diagnostic value of targeted next-generation sequencing for infectious diseases (review). Mol. Med. Rep. 30, 153. doi: 10.3892/mmr.2024.13277. PMID: 38963022

[B6] Chesney R. W. Carpenter M. A. Moxey-Mims M. Nyberg L. Greenfield S. P. Hoberman A. . (2008). Randomized intervention for children with vesicoureteral reflux (RIVUR): background commentary of RIVUR investigators. Pediatrics 122, S233–S239. doi: 10.1542/peds.2008-1285c. PMID: 19018047 PMC4336951

[B7] Davison F. Chapman J. Mickiewicz K. (2020). Isolation of L-form bacteria from urine using filtration method. J. Vis. Exp. 160. doi: 10.3791/61380. PMID: 32597873

[B8] Flores-Mireles A. L. Walker J. N. Caparon M. Hultgren S. J. (2015). Urinary tract infections: epidemiology, mechanisms of infection and treatment options. Nat. Rev. Microbiol. 13, 269–284. doi: 10.1038/nrmicro3432. PMID: 25853778 PMC4457377

[B9] Gohil S. K. Septimus E. Kleinman K. Varma N. Avery T. R. Heim L. . (2024). Stewardship prompts to improve antibiotic selection for urinary tract infection: the INSPIRE randomized clinical trial. Jama 331, 2018–2028. doi: 10.1001/jama.2024.6259. PMID: 38639723 PMC11185978

[B10] He S. Liu H. Hu X. Zhao J. Liang J. Zhang X. . (2024). Exploring the clinical and diagnostic value of metagenomic next-generation sequencing for urinary tract infection: a systematic review and meta-analysis. BMC Infect. Dis. 24, 1000. doi: 10.1186/s12879-024-09914-9. PMID: 39294577 PMC11412013

[B11] Hermanides H. S. Hulscher M. E. Schouten J. A. Prins J. M. Geerlings S. E. (2008). Development of quality indicators for the antibiotic treatment of complicated urinary tract infections: a first step to measure and improve care. Clin. Infect. Dis. 46, 703–711. doi: 10.1086/527384. PMID: 18230045

[B12] Jia K. Huang S. Shen C. Li H. Zhang Z. Wang L. . (2023). Enhancing urinary tract infection diagnosis for negative culture patients with metagenomic next-generation sequencing (mNGS). Front. Cell. Infect. Microbiol. 13. doi: 10.3389/fcimb.2023.1119020. PMID: 36936777 PMC10020507

[B13] Kolvenbach C. M. Shril S. Hildebrandt F. (2023). The genetics and pathogenesis of CAKUT. Nat. Rev. Nephrol. 19, 709–720. doi: 10.1038/s41581-023-00742-9. PMID: 37524861

[B14] Kranz J. Bartoletti R. Bruyère F. Cai T. Geerlings S. Köves B. . (2024). European association of urology guidelines on urological infections: summary of the 2024 guidelines. Eur. Urol. 86, 27–41. doi: 10.1016/j.eururo.2024.03.035. PMID: 38714379

[B15] Mattoo T. K. Shaikh N. Nelson C. P. (2021). Contemporary management of urinary tract infection in children. Pediatrics 147, e2022059259. doi: 10.1542/peds.2020-012138. PMID: 33479164

[B16] McAteer J. Lee J. H. Cosgrove S. E. Dzintars K. Fiawoo S. Heil E. L. . (2023). Defining the optimal duration of therapy for hospitalized patients with complicated urinary tract infections and associated bacteremia. Clin. Infect. Dis. 76, 1604–1612. doi: 10.1093/cid/ciad009. PMID: 36633559 PMC10411929

[B17] Millner R. Becknell B. (2019). Urinary tract infections. Pediatr. Clin. North. Am. 66, 1–13. doi: 10.1016/j.pcl.2018.08.002. PMID: 30454735

[B18] Nartey L. K. Mikhael A. Pětrošová H. Yuen V. Kibsey P. Pekcan M. . (2024). A lipidomics-based method to eliminate negative urine culture in general population. J. Clin. Microbiol. 62, e0081924. doi: 10.1128/jcm.00819-24. PMID: 39283074 PMC11481538

[B19] Nelson Z. Aslan A. T. Beahm N. P. Blyth M. Cappiello M. Casaus D. . (2024). Guidelines for the prevention, diagnosis, and management of urinary tract infections in pediatrics and adults: a WikiGuidelines Group consensus statement. JAMA Netw. Open 7, e2444495. doi: 10.1016/b978-0-323-06969-4.00024-6. PMID: 39495518

[B20] Oreskovic N. M. Sembrano E. U. (2007). Repeat urine cultures in children who are admitted with urinary tract infections. Pediatrics 119, e325–e329. doi: 10.1542/peds.2006-1134. PMID: 17272596

[B21] Price T. K. Dune T. Hilt E. E. Thomas-White K. J. Kliethermes S. Brincat C. . (2016). The clinical urine culture: enhanced techniques improve detection of clinically relevant microorganisms. J. Clin. Microbiol. 54, 1216–1222. doi: 10.1128/jcm.00044-16. PMID: 26962083 PMC4844725

[B22] Puri P. Friedmacher F. Farrugia M. K. Sharma S. Esposito C. Mattoo T. K. . (2024). Primary vesicoureteral reflux. Nat. Rev. Dis. Primers 10, 75. doi: 10.1038/s41572-024-00560-8. PMID: 39389958

[B23] Rubin R. H. Shapiro E. D. Andriole V. T. Davis R. J. Stamm W. E. (1992). Evaluation of new anti-infective drugs for the treatment of urinary tract infection. Infectious Diseases Society of America and the Food and Drug Administration. Clin. Infect. Dis. 15, S216–S227. doi: 10.1093/clind/15.supplement_1.s216. PMID: 1477233

[B24] Simões E. S. A. C. Oliveira E. A. Mak R. H. (2020). Urinary tract infection in pediatrics: an overview. J. Pediatr. (Rio J) 96 Suppl 1, 65–79. doi: 10.1016/j.jped.2019.10.006 31783012 PMC9432043

[B25] Timm M. R. Russell S. K. Hultgren S. J. (2025). Urinary tract infections: pathogenesis, host susceptibility and emerging therapeutics. Nat. Rev. Microbiol. 23, 72–86. doi: 10.1038/s41579-024-01092-4. PMID: 39251839 PMC13194463

[B26] Wagenlehner F. M. E. Bjerklund Johansen T. E. Cai T. Koves B. Kranz J. Pilatz A. . (2020). Epidemiology, definition and treatment of complicated urinary tract infections. Nat. Rev. Urol. 17, 586–600. doi: 10.1038/s41585-020-0362-4. PMID: 32843751

[B27] Yin Y. Zhu P. Guo Y. Li Y. Chen H. Liu J. . (2024). Enhancing lower respiratory tract infection diagnosis: implementation and clinical assessment of multiplex PCR-based and hybrid capture-based targeted next-generation sequencing. EBioMedicine 107, 105307. doi: 10.1016/j.ebiom.2024.105307. PMID: 39226681 PMC11403251

[B28] Zhan Z. Lin X. Li G. Zeng J. Su D. Liao J. . (2023). Renal abscess complicating acute pyelonephritis in children: two cases report and literature review. Med. (Baltimore) 102, e36355. doi: 10.1097/md.0000000000036355. PMID: 38050281 PMC10695508

