# Supplementary figures and images for: Efficacy of hc-tNGS for pathogen identification for pediatric cUTIs: a real-world observational study
Source: Front Cell Infect Microbiol. 2026 May 8;16:1826277. doi: 10.3389/fcimb.2026.1826277 (PMC13194379; doi:10.3389/fcimb.2026.1826277)

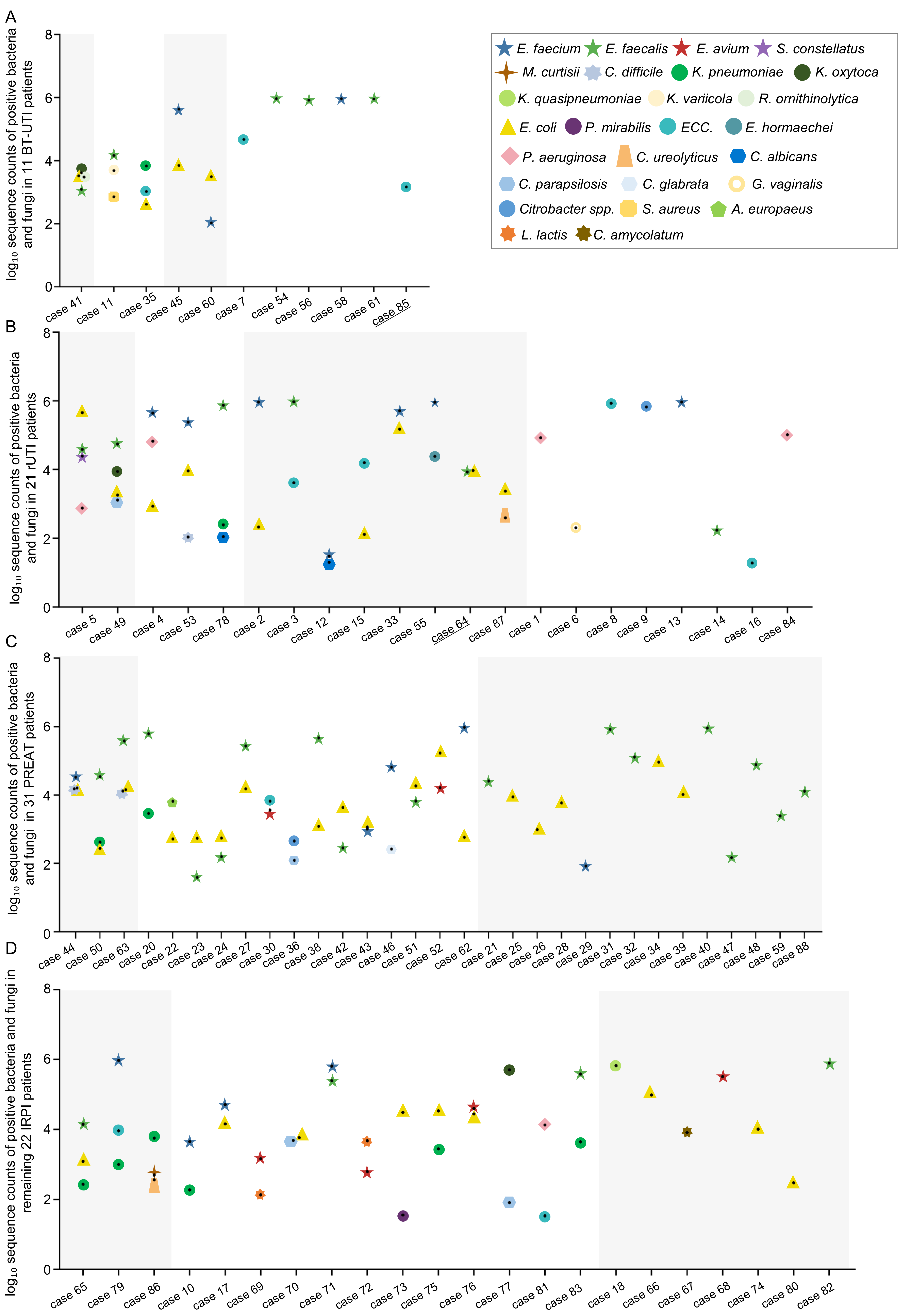

Supplement: Supplementary Figure 1 — Sequence counts (log10 scale) of positive bacteria and fungi in 85 hc-tNGS positive pediatric cUTI patients enrolled in this study, stratified according to the criteria for cUTI: breakthrough UTI (BT-UTI) (A), recurrent UTI (rUTI) (B), poor response to empirical antimicrobial therapy (PREAT) (C), and invasive renal parenchymal infection (IRPI) (D). The x-axis (Case + number) indicates the patient identifier. Underlined case numbers (excluding the IRPI group) denote patients who also presented with IRPI. Different colored symbols represent distinct bacterial or fungal species. The black dot at the center of each symbol denotes the exact log10 value of the sequence count for the corresponding microorganism. E. faecium, Enterococcus faecium; E. faecalis, Enterococcus faecalis; E. avium, Enterococcus avium; S. constellatus, Streptococcus constellatus; M. curtisii, Mobiluncus curtisii; C. difficile, Clostridioides difficile; K. pneumoniae, Klebsiella pneumoniae; K. oxytoca, Klebsiella oxytoca; K. quasipneumoniae, Klebsiella quasipneumoniae; K. variicola, Klebsiella variicola; R. ornithinolytica, Raoultella ornithinolytica; E. coli, Escherichia coli; P. mirabilis, Proteus mirabilis; ECC, Enterobacter cloacae complex; E. hormaechei, Enterobacter hormaechei; P. aeruginosa, Pseudomonas aeruginosa; C. ureolyticus, Campylobacter ureolyticus; C. albicans, Candida albicans; C. parapsilosis, Candida parapsilosis; C. glabrata, Candida glabrata; G. vaginalis, Gardnerella vaginalis; Citrobacter spp., Citrobacter species; S. aureus, Staphylococcus aureus; A. europaeus, Actinomyces europaeus; L. lactis, Lactococcus lactis; C. amycolatum, Corynebacterium amycolatum. [file Image1.tif]
